# 

**Published:** 1851-04-01

**Authors:** 


We must again crave the kind indulgence of our numerous correspondents. We hope, in our next number, to satisfy all parties, and to notice everything that has been forwarded to the journal. Many American periodicals are transmitted to us which we are compelled to refuse, in consequence of the postage amounting to a large sum, varying between 8*s*., 9*s*., 10*s*., and 18*s*. Our correspondents abroad should see to this.

We are compelled to reserve, until July, our usual notice of books, exchanges, &c.

